# The factor XI/XIa antibody abelacimab combined with enoxaparin inhibits filter clotting in hemodialysis circuits ex vivo

**DOI:** 10.1007/s11239-024-03059-x

**Published:** 2024-11-16

**Authors:** Juergen Grafeneder, Gesche Langer, Christian Schoergenhofer, Farsad Eskandary, Bernd Jilma, Yasser Khder, Katarina D. Kovacevic Miljevic

**Affiliations:** 1 Department of Clinical Pharmacology, Medical University of Vienna, Vienna, Austria; 2 Department of Emergency Medicine, Medical University of Vienna, Vienna, Austria; 3 Department of Nephrology and Dialysis, Division of Medicine III, Medical University, Vienna, Austria; 4 MYRA LSS, Rosenau, France

**Keywords:** Abelacimab, Coagulation, Factor XI, Hemodialysis, Thromboelastometry

## Abstract

Drugs targeting factor XI may offer an alternative to heparin for preventing blood clotting in extracorporeal circulation. We investigated the effects of abelacimab, a novel monoclonal antibody targeting factor XI. We collected whole blood samples into two bags (each 240 ml, control group: enoxaparin 1.2 mg, treatment group: enoxaparin 1.2 mg plus abelacimab 5 mg) and circulated in a hemodialysis device for up to 3 h. We performed whole blood aggregation and thromboelastometry at several time points. Time to filter clotting was the primary endpoint. We included 10 volunteers. Each volunteer’s blood was split into two bags (containing enoxaparin +/- abelacimab) and used simultaneously on two hemodialysis devices. The treatment group’s time to filter clotting was significantly prolonged (treatment: 180 min, IQR 180–180 vs. control: 120 min, IQR 97–147, *p* < 0.001), and the transmembrane pressure was significantly lower at the end of the circuit flow (treatment: 13 mmHg vs. control: 65 mmHg, *p* = 0.001). Fibrinogen levels and median platelet counts were preserved. Platelet aggregation was better preserved in the treatment group for ristocetin (*p* = 0.015), thrombin receptor activating peptide (*p* = 0.015), and arachidonic acid (*p* = 0.001). Thromboelastometry showed prolonged clotting times in the treatment group at the end of the experiment (INTEM, *p* < 0.001; HEPTEM, *p* = 0.001). Abelacimab prolonged the time to filter clotting in this ex vivo model of hemodialysis. This is an aggressive model due to the frequent re-circulation of blood and a lack of endothelial cells. These data provide support for testing abelacimab in patients on hemodialysis.

**Supplementary Information:**

The online version contains supplementary material available at 10.1007/s11239-024-03059-x.

## Key points

Extracorporeal circulation involves blood contact with a synthetic surface, leading to the initiation of the coagulation cascade.

Drugs targeting factor XI may be an alternative to heparin in extracorporeal circulation.

We tested the addition of abelacimab, a monoclonal antibody targeting factor XI, to enoxaparin.

Abelacimab prolonged the time to filter clotting in an ex vivo hemodialysis circuit.

Our data suggest abelacimab’s potential as a therapeutic option.

**Supplementary Information:**

The online version contains supplementary material available at 10.1007/s11239-024-03059-x.

## Introduction

Extracorporeal circulation exposes blood to an artificial surface, which activates the coagulation cascade. Anticoagulation reduces the risk of thrombosis, but increases the risk of bleeding. The most common example of extracorporeal circuits in human medicine is hemodialysis, with patients already at high risk of bleeding [1, 2]. The current standard of care to prevent extracorporeal circuit coagulation in most dialysis centers is using intravenous low molecular weight heparin on an off-label use basis [3]. 

Factor XI (FXI) is one potential target for new therapeutics. It is activated by the contact activation pathway (factor XII) or thrombin. FXI is essential for forming and stabilizing a thrombus, but shows mild hemostatic activities [4]. Congenital FXI deficiency is rarely associated with spontaneous bleeding [5], and FXI plasma levels correlate poorly with bleeding risk [6]. A threshold of 20% of FXI activity is defined as severe FXI deficiency since individuals below 20% show an increased risk of bleeding [7, 8]. Of studies comparing various factor XI inhibitors with enoxaparin after total knee arthroplasty, factor XI inhibitors not just significantly reduced the risk of VTE (risk ratio 0.59 (95%CI 0.37–0.94), but also the bleeding risk (risk ratio 0.41 (95% CI 0.19–0.92)) [9]. Several studies investigated the therapeutic inhibition of Factor XI [10]. Two studies investigated patients with end-stage renal disease, and their results were promising [11, 12]. Thus, inhibiting FXI seems to be a promising target for new therapeutics.

Abelacimab is a fully humanized monoclonal antibody, that binds to the catalytic domain of both FXI zymogen and its activated form (FXIa) with equal potency, locking it into the inactive precursor and preventing its activation [13]. 

We hypothesized that adding abelacimab to hemodialysis (HD) circuits prolongs the time to circuit clotting.

## Materials and methods

This study was performed at the Department of Clinical Pharmacology at the Medical University of Vienna. The Ethics Committee of the Medical University of Vienna approved this study, which was conducted following the Declaration of Helsinki. All subjects gave their written and oral informed consent.

Eligibility criteria for healthy volunteers included age between ≥ 18 and ≤ 60 years, normal findings in their medical history, and physical examination. We excluded volunteers with known hepatitis B or C virus or human immunodeficiency virus infection, blood donations done 1 month before this study, or relevant history that may interfere with the aim of the study. Furthermore, patients with anemia (< 12 g/dl hemoglobin for females and < 13.5 g/dl hemoglobin for males) were excluded. The use of medication within two weeks before the start of the study, which the investigator considered relevant to the study’s objectives, was also considered exclusion criteria. The full list of inclusion/exclusion criteria can be found in the Supplement (Table S1).

### Blood collection and sampling

A total of 480 mL blood was collected from each volunteer by *de novo* venipuncture and divided into two bags (240 mL whole blood), pre-filled with either 1.2 mg enoxaparin (control group) or 1.2 mg enoxaparin plus 5 mg abelacimab (treatment group). We chose enoxaparin to prevent blood from clotting between blood sampling and the start of the experiment. Furthermore, enoxaparin is the extracorporeal circuit anticoagulation recommended by the European Best Practice Guidelines for hemodialysis. This dosage is based on previous studies [14], which showed that 5 µg/mL prevents the circuit from clotting within 120 min. Blood samples for coagulation and platelet function assays were collected at baseline (before circuit initiation) and 15 min, 30 min, 1 h, 2 h, and 3 h after circuit initiation. Blood was drawn directly from the blood bag at baseline and from the closed circuits via a port on the circuits for further time points. The conditions under which the study was conducted were the same for both circuits (treatment and control group).

### Hemodialysis

We chose an ex vivo system because it is a more aggressive model. A higher rate of blood circulation results in higher coagulation factor activity than in the clinical situation. Our ex vivo model was also used for proof of concept with osocimab, which provided a benchmark for the current testing of abelacimab under this challenging condition, leading to a more robust rationale for future in vivo trials.

The extracorporeal circuits comprised Nikkiso Hemodialysis devices (DBB 07), Filters (EF-02D), and F.M. S.p.A bloodline (AV03-B). Before the circuits were filled with whole blood, they were pre-filled with 0.9% NaCl.

The ultrafiltration fraction was set to zero to avoid hemoconcentration. The dialysate flow was set to 100 mL/min single pass. The dialysate AC-F313/2 (Fresenius) was used in the study. A picture of the experimental setup can be found in the supplement material (Figure SF1).

The efficacy of the HD circuit was assessed by quantifying potassium concentrations at baseline and the end of each HD run.

Transmembrane pressure was recorded at baseline and 30, 60, 120, 150, and 180 min after the start of the experiment or at the time of filter clotting (end of dialysis), whichever happened first. Filter clotting was defined as a sudden rise in transmembrane pressure to a level where the dialysis machine stopped automatically. A transmembrane pressure of 50 mmHg was defined as the cut-off for clotting.

### Analysis of global coagulation parameters

Global coagulation tests (fibrinogen, platelet count, activated partial thromboplastin time (aPTT), prothrombin time) were performed according to standardized, routinely used laboratory assays in ISO-certified laboratories of the hospital. A detailed description can be found in a previous publication [14]. 

### Whole blood platelet aggregometry

We used the multiple electrode aggregometry on an impedance aggregometer (Multiplate Analyzer; Dynabyte) to determine whole blood aggregation. A detailed description can be found in a previous publication [15]. The results were quantified in arbitrary aggregation units (AU x min) or units (U) as the area under the curve of the aggregation tracing.

### Thromboelastometry

We used a rotational thromboelastometry (ROTEM Coagulation Analyzer, Pentapharm, Munich, Germany) to analyze clot formation and subsequent fibrinolysis. Whole blood samples were collected in 3.8% sodium citrate. We used the INTEM and HEPTEM tests. INTEM measures coagulation via activation of the intrinsic system (phospholipid and ellagic acid). HEPTEM activates the coagulation the same way as INTEM but uses lyophilized heparinase for neutralizing heparin.

### Statistical analysis

We did not perform an a priori sample size calculation because of the unknown magnitude of the effect size. Categorical variables are summarized as counts (n) and frequencies (%) and are compared using Fisher’s exact test or Chi-square. Continuous variables are expressed as mean, standard deviation (±) or median, and interquartile range (IQR). They are compared using Mann-Whitney U Test or t-Test as appropriate.

The primary endpoint, time to filter clotting, was compared using a Mann-Whitney U-Test. Secondary endpoints (including transmembrane pressure, TMP) are used for hypothesis generation. We used binominal logistic regression analysis to estimate the effect of additional abelacimab on circuit clotting within 180 min.

In case filter clotting did not occur after 180 min, a value of 180 min was assigned for conservative statistical comparisons. If a value exceeded the upper measurable limit, the maximal value plus one was assigned (e.g., > 180 s for activated partial thromboplastin time, a value of 181 s was assigned).

Two-sided p-values of < 0.05 indicated statistical significance. No imputation for missing data was performed. R statistical software environment (R Foundation for Statistical Computing, Vienna, Austria, http://www.R-project.org, version 3.6.2) was used for all analyses.

## Results

We included 9 male and 1 female (mean age: 37 ± 11 years) volunteers. Table 1 shows baseline characteristics.

**Table 1:** Baseline characteristics

Variable	
Total, n	10
Gender female, n (%)	1 (10%)
Age [years], mean (SD)	37 (11)
Caucasian, n (%)	10 (100)
Hemoglobin [g/dL], mean (SD)	15 (0.7)
Platelet count [G/L], mean (SD)	228 (37)
White blood cells [G/L], mean (SD)	5.8 (1.8)
Thromboplastin time* [%], mean (SD)	94.0 (16.7)
INR, mean (SD)	1.0 (0.1)
aPTT [s], mean (SD)	36.3 (7.2)
Fibrinogen** [mg/dL], mean (SD)	264 (60)

*Method by Owren, reference level: 70-130%; **method by Clauss, normal reference level: 200-400 mg/dL; aPTT… activated Partial Thromboplastin Time, normal reference level: 27–41 s

### Primary endpoint

**Fig. 1:**
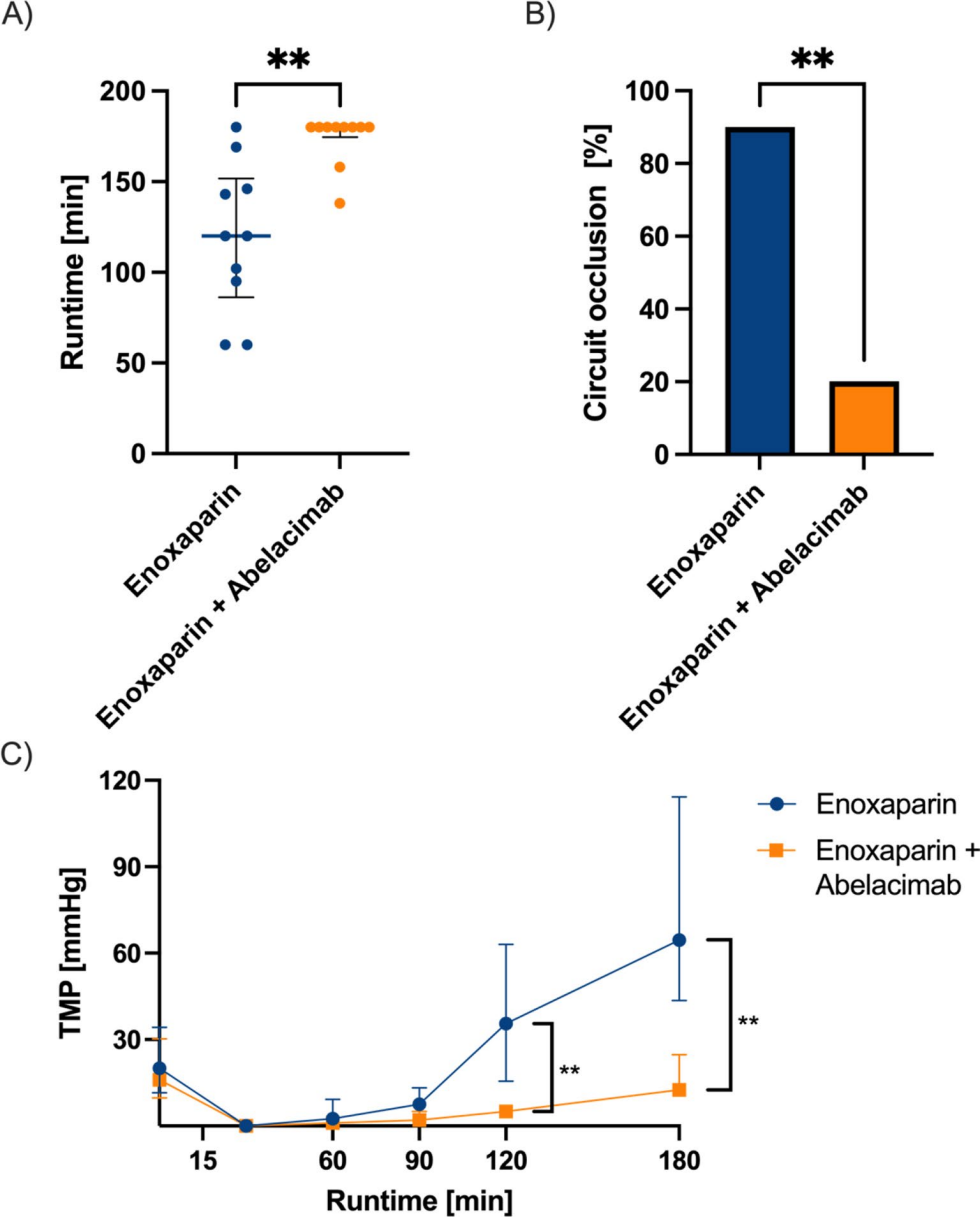
Comparison of runtime (**A**), circuit occlusion (**B**), and transmembrane pressure (**C**) between the control group (Enoxaparin 1.2 mg, *n*=10) and the treatment group (Enoxaparin 1.2 mg plus Abelacimab 5 mg, *n*=10). Thrombin formation occurred in every circuit but did not reach the occlusion threshold within the predefined maximum runtime (i.e., 180 min). Data are shown in median and interquartile range (**A**, **C**) and frequency (**B**). ** *p* < 0.001. TMP… transmembrane pressure

The time to filter clotting was significantly longer in the treatment group using enoxaparin together with abelacimab when compared to enoxaparin alone (treatment: median 180 min, IQR 180–180 vs. control: median 120 min, IQR 95–146, *p* = 0.002, Fig. 1A). Adding abelacimab to the circuit significantly reduced the odds of circuit clotting within 180 min with a crude odds ratio of 0.028 (95% CI 0.002–0.367, *p* = 0.007, Fig. 1B). Abelacimab reduced the transmembrane pressure at the end of the dialysis procedure (treatment: median 13 mmHg, IQR 11–24 vs. control: median 65 mmHg, IQR 48–114, *p* = 0.001, Fig. 1C). The treatment group experienced only two instances of premature termination before reaching the maximum 180-minute runtime. This was due to a blood shortage problem: In three of the first five circuits (two in the intervention group and one in the control group), the dialysis machine alerted because of blood shortage. We switched to a different machine (the exact same model) for the rest of the experiment since we did not find any leakage or clear explanation for this. Afterwards, this phenomenon did not reoccur. The blood shortage itself did not cause an increase in the TMP. In the intervention group, the TMP at the end of the circuit flow was 24 mmHg and 27 mmHg, respectively. In the control group, the TMP was 63 mmHg at the end of the circuit flow (See Table 2).

**Table 2:** Coagulation parameters were measured at the experiment’s end for the control (1.2 mg enoxaparin) and treatment (1.2 mg enoxaparin + abelacimab 5 mg) group. Data are shown in the median and interquartile ranges

Variable	Control	Treatment	*p*-value
**Hemodialysis**			
Runtime [min]	120 (95 - 146)	180 (180 - 180)	**0.002**
TMP [mmHg]	57 (30 - 114)	12.5 (11 - 19)	**0.001**
**Global coagulation parameters**			
Fibrinogen [mg/dL]	178 (154 - 201)	206.5 (189 - 224)	0.064
Platelets [G/L]	106 (95 - 119)	135 (127 - 149)	**0.035**
aPTT [s]	40.6 (37.2 - 42.1)	108.2 (103.7 - 127.8)	**< 0.001**
PT* [%]	80 (72 - 96)	84 (73 - 108)	0.579
INR	1.1 (1.0 - 1.2)	1.1 (1.0 - 1.2)	0.684
Anti-Xa [IU/mL]	0.65 (0.55 - 0.68)	0.58 (0.49 - 0.69)	0.631
**Platelet Aggregation**			
Multiplate			
ADP [Units]	10.5 (10 - 15)	15 (11 - 15)	0.247
ASPI [Units]	16.5 (16 - 21)	23 (22 - 33)	**0.035**
RISTO [Units]	2 (0 - 13)	21.5 (16 - 31)	**0.015**
TRAP [Units]	17 (13 - 18)	28.5 (25 - 44)	**0.019**
**ROTEM**			
INTEM CT [s]	158.5 (135 - 175)	401.5 (331 - 431)	**< 0.001**
INTEM CFT [s]	156.5 (128 - 183)	120 (117 - 152)	0.063
INTEM MCF [mm]	50 (57 - 55)	56 (50 - 56)	0.190
INTEM ML [%]	0 (0 - 0)	0 (0 - 0)	
HEPTEM CT [s]	158.5 (122 - 166)	320 (288 - 402)	**0.001**
HEPTEM CFT [s]	165 (123 - 190)	110 (104 - 132)	**0.023**
HEPTEM MCF [mm]	50 (47– 56)	54 (52– 57)	0.222
HEPTEM ML [%]	0 (0 - 0)	0 (0 - 0)	

*Method by Owren, reference level: 70-130%; TMP… transmembrane pressue; aPTT… activated partial thromboplastin time; PT… prothrombin time; ADP… adenosine diphosphate; ASPI… arachidonic acid; RISTO… ristocetin; TRAP… thrombin receptor-activating peptide 6; ROTEM… rotational thromboelastometry; CT… clotting time; CFT… clot formation time; MCF… maximum clot firmness; ML… maximum lysis

### Secondary endpoints

**Fig. 2:**
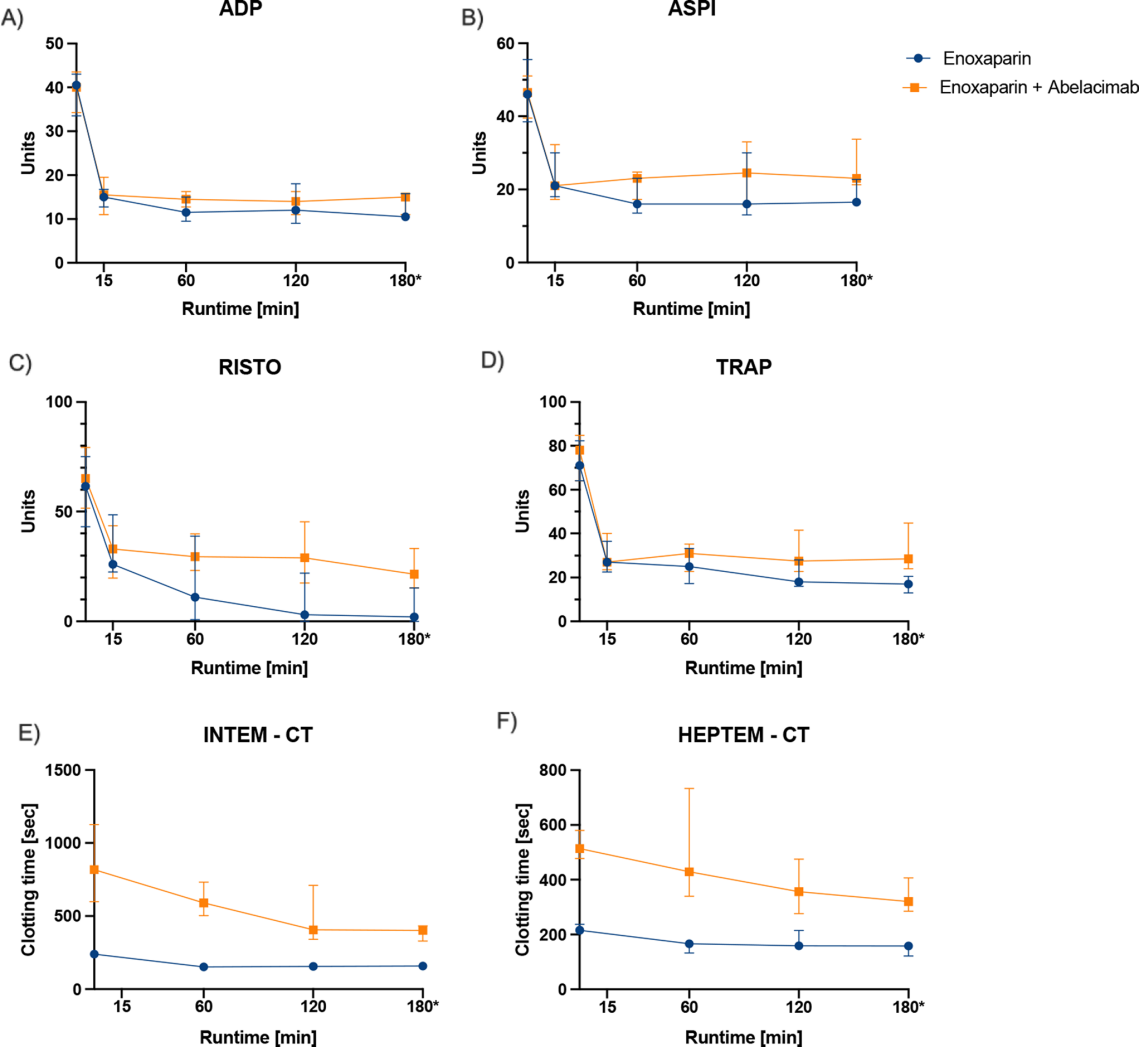
Results of the platelet aggregation measured by the Multiplate Analyzer (**A**-**D**, (Dynabyte) and thromboelastography ROTEM Coagulation Analyzer (**E**-**F**, Pentapharm, Munich, Germany) for the control group (Enoxaparin 1.2 mg, *n*=10, in blue) and the treatment group (Enoxaparin 1.2 mg plus Abelacimab 5 mg, *n*=10, in orange). The INTEM module measures coagulation via activation of the intrinsic system (phospholipid and ellagic acid). HEPTEM activates the coagulation the same way as INTEM but uses lyophilized heparinase for neutralizing heparin. Data are shown as median and interquartile range. ADP… adenosine diphosphate; ASPI… arachidonic acid; RISTO… ristocetin; TRAP… thrombin receptor-activating peptide; CT… Clotting time

The treatment group showed a higher residual aggregation to arachidonic acid, ristocetin, and thrombin receptor-activating peptide (*p* = 0.035, *p* = 0.015, and *p* = 0.019, respectively; Fig. 2A-D). Clotting times were prolonged in thromboelastometry (Fig. 2E-F). For INTEM, the median clotting time at the end of the experiment was 402 s (IQR 331–431) vs. 159 s (IQR 135–175) for the treatment group and the control group, respectively (*p* < 0.001). The same was true for the HEPTEM measurement (320 s, IQR 288–402 vs. 159 s, IQR 122–166, *p* < 0.001). The maximum clot firmness did not differ between the groups for INTEM and HEPTEM (*p* = 0.190, *p* = 0.222). There was no sign of fibrinolysis in either group.

Abelacimab partially influenced global coagulation tests at the end of the experiment. The median aPTT was significantly longer in the abelacimab group (treatment: median 108 s, IQR 104–128 vs. control: median 41 s, IQR 37–42, *p* < 0.001). Furthermore, median platelet numbers were preserved (treatment: median 135 G/L, IQR 127–149 vs. control: median 106 G/L, IQR 95–119, *p* = 0.035). The median platelet count at the start of the experiment was 152 G/L (IQR 135–170) for the treatment group and 140 G/L (IQR 126–166) for the control group, which did not differ significantly (*p* = 0.481, Table S2, Supplement). The median PT time (treatment: 84%, IQR 73–108 vs. control: 80%, IQR 72–96%, *p* = 0.105) and the median fibrinogen level (treatment: 206.5 mg/dL, IQR 189–224 vs. control: 178 mg/dL, IQR 154–201, *p* = 0.063) did not show a significant difference between the two groups. Anti-Xa (treatment: median 0.58, IQR 0.49–0.69 vs. control: median 0.65, IQR 0.55–0.68, *p* = 0.631) and hemoglobin levels (treatment: 12.5 g/dL, IQR 11.5–13.0 vs. control: median 12.6, IQR 11.9–13.1, *p* = 0.623) did not differ significantly either. The median potassium level across all circuits decreased by 0.35 mmol/L (from a median of 3.25, IQR 3.2–3.35 to a median of 2.9, IQR 2.9–3, Table S2, Supplement).

## Discussion

The present study aimed to investigate the potential effects of abelacimab in an ex-vivo model of extracorporeal circulation. Our results demonstrated promising outcomes following adding abelacimab to enoxaparin. This resulted in a significant reduction in filter clotting, as indicated by the transmembrane pressure (13 mmHg vs. 65 mmHg). This decrease in filter clotting was also associated with a longer runtime (180 min vs. 120 min). Additionally, the group that received abelacimab exhibited higher platelet count and residual aggregation.

The transmembrane pressure is measured between the filtrate and permeate sides of the membrane [16]. Increased transmembrane pressure is associated with a stepwise clogging of the filter, resulting in reduced hemodialysis performance [17]. In the control group (enoxaparin 1.2 mg without abelacimab), only one circuit reached the 180 min runtime. However, this circuit was very unstable for the last 30 min, and the transmembrane pressure reached 48 mmHg, with 50 mmHg being the cut-off for clotting. None of the circuits with abelacimab showed any sign of a sudden increase in transmembrane pressure. All other circuits in the control group stopped earlier due to clotting, except for one, which stopped due to the lack of blood in the circuit, which is more of a technical issue.

Hemostatic disorders are commonly observed in patients undergoing dialysis, presenting as both thrombosis and bleeding tendencies [18]. One reason for the hypercoagulable state is the coagulation and platelet activation within the extracorporeal HD machine [19]. Therefore, these patients are at high risk for thromboembolic complications [20]. In the abelacimab treatment group, prolonged clotting time for the INTEM and HEPTEM with a prolonged aPTT suggests sufficient anticoagulation. On the other hand, patients with end-stage chronic kidney disease show an increased risk of bleeding, even in the absence of anticoagulation [21–24], and show reduced platelet aggregation [20]. Unfortunately, there is a lack of evidence for randomized trials using direct oral anticoagulants in patients with kidney failure. However, data suggests an increase in bleeding events or no difference compared to warfarin, except for apixaban [25–28]. Factor XI inhibition is hypothesized to reduce the risk of thrombosis while having a minimal effect on hemostasis [29–31]. Our data support this assumption. The multiplate aggregation results at the end of the hemodialysis likely reflect a better-preserved platelet function. These results need further confirmation in vivo because data from gruticibart show unaltered in vivo platelet activation [32]. The differences in our results may be due to the shorter interval between applying the factor XI inhibitor, the platelet function measurement, and the different platelet function measurement methods. Furthermore, the ex-vivo nature of our experiment leads to higher shear stress, higher platelet consumption (due to the lack of platelet generation), and more platelet activation compared to in vivo [32]. 

Abelacimab did not influence the maximum clot firmness, and there was no sign of fibrinolysis. The aPTT was significantly prolonged, and, as expected, the PT was unaffected. Both fibrinogen and platelets did not show a sign of consumption. This is especially important in hemodialysis patients since the increased cardiovascular morbidity and mortality correlate with platelet reactivity [33, 34]. 

Several studies have investigated other factors, such as XI inhibitors in hemodialysis patients. Lorentz et al. investigated AB023 (gruticibart) in 24 patients with end-stage renal disease undergoing heparin-free hemodialysis [12]. AB023 was well tolerated and reduced clotting within the dialyzer compared to placebo (rate of high-grade dialyzer clotting on study day 3: 87.5% in the placebo group, 62.5% in the 0.5 mg/kg gruticibart group). The maximum increase in aPTT was observed 3 to four hours after administration of gruticibart on study day 1 (96.0 ± 4.1 s in the 0.5 mg/kg gruticibart group). In comparison, the clotting rate in our study was 20% for the treatment group and 90% for the control group. The aPTT at the end of the experiment (i.e., up to 180 min) was 108.5 s (± 28.1) in our treatment group (i.e., enoxaparin plus abelacimab) and 39.4 s (± 3.6) in our control group.

Weitz et al. investigated osocimab in a phase 2b double-blind, placebo-controlled trial in patients with kidney failure undergoing hemodialysis [35]. Osocimab was well tolerated by the 686 participants and showed a low risk of bleeding. Clotting of the dialysis circuit was scored from 0 (no clot) to 3 (fully clotted system requiring interruption of hemodialysis session). Circuit clotting of 2 (intermediate between 1, i.e., trace of clotting, and 3, i.e., fully clotted system) or 3 was reduced (41.3% in the placebo group, 29.3 in the lower-dose osocimab group, and 27.3% in the higher-dose osocimab group). The aPTT was approximately 1.4-fold higher compared to the baseline four days after the first dose in the higher-dose osocimab group. In comparison, the increase in median aPTT was 3.1-fold in our treatment group (end of study: 108.2 s, IQR 103.7–127.8 vs. baseline: 34.5 s, IQR 30.5–35.6).

Winkelmeyer et al. [36] investigated fesomersen in a phase 2 randomized controlled trial. In 307 patients with kidney failure on hemodialysis, fesomersen produced a dose-dependent factor XI reduction with similar rates of major bleeding compared to the placebo (6.5–3.9% in the fesomersen group, depending on dosage vs. 4.0% in the placebo group). Clotting was quantified using the semiquantitative clotting score (from 0, no visible clotting, to 3, fully clotted extracorporeal system, resulting in an interruption of the hemodialysis session). The rate of ≥ 1 event of a score 2 (intermediate between 1, traces of coagulation, and 3) or 3 did not differ significantly between the placebo (29.3%) and the three fesomersen groups (40 mg: 22.1%, 80 mg: 30.4%, and 120 mg: 21.1%). However, the factor XI reduction by fesomersen was associated with the circuit-clotting scores of 2 or 3, assessed by logistic regression.

Other indications are investigated in addition to hemodialysis patients. Pfeffer et al. tested gruticibart in a prospective, single-arm study [32]. A total of 22 ambulatory cancer patients received gruticibart after undergoing central line placement. During the study period (days 0–30), the catheter thrombus rate was reduced when measured by ultrasound (40% in the control group vs. 12% in the gruticibart group). Based on the effects observed in our in vitro study, it will be interesting to see whether abelacimab will perform similarly under these clinical conditions.

Meyer and colleagues performed a study using an ex vivo pediatric extracorporeal membrane oxygenation circuit [37]. Blood was collected from 13 volunteers and either spiked with unfractionated heparin alone or in combination with gruticibart. The experimental circulation time was either 6 h or until device failure was caused by clot formation. Occlusion was present in 3/8 (60%) of the circuits treated with unfractionated heparin alone. Interestingly, in the group including gruticibart, all circuits (*n* = 5) sustained. In our study, circuit occlusion appeared in 90% (9/10) of the control group. In the treatment group, no circuit had to be stopped due to circuit occlusion. Two circuits in the treatment group had to be stopped prematurely because of blood shortage. The TMP was 24 mmHg and 27 mmHg, respectively, at the end of the circuit flow.

We added the low-molecular-weight heparin (LMWH) enoxaparin (5 µg/mL) to all blood bags. This dose was based on previous studies, which showed that this dosage prevents the circuits from clotting within 120 min [14]. It is difficult to compare the enoxaparin concentration of the experiment to the enoxaparin concentration used in hemodialysis. The use of low-molecular-weight heparin to reduce clotting of the extracorporeal system is recommended [3, 38]. In our center, it is common practice to administer 4.000 IE of enoxaparin, irrespective of weight. However, as a recently published Cochrane review pointed out, the overall evidence for anticoagulation to prevent clot formation is sparse [39]. Thrombin antithrombin complexes still increase 2-3-fold after a bolus of 75U/kg LMWH [40]. An anti-Xa activity of > 0.4 IU/ml significantly inhibited clotting after 4 h of dialysis [41]. A study by Xu et al. [42] reported that an anti-Xa activity of > 0.2 IU/ml is crucial for the coagulation grade of the filter and line during intermittent venovenous hemofiltration lasting 6 h. After administering the predefined amount of enoxaparin, a level of > 0.4 IU/ml was found in all the whole blood bags used in our experiment.

There are certain limitations to this study. Caution is warranted to extrapolate these in vitro results to the biology of intact organisms. One important difference is the high frequency of blood recirculation without the ability for the blood to recover. Furthermore, interactions between blood components and the endothelial system are missed. This leads to a more aggressive model resulting in more pronounced clotting than in the clinical situation, necessitating higher than usual concentrations of anti-Xa activity to prevent the circuit from occluding within 2 h. Although a clinical situation might yield different results, abelacimab currently is not approved by the US Food and Drug Administration or the European Medicines Agency to be used in hemodialysis patients. Given the in vitro nature of this study, bleeding risks cannot be assessed. Additional studies are needed to address whether abelacimab alone can be used as an alternative to heparin for hemodialysis.

In conclusion, our study shows that abelacimab not only prolonged the time to filter clotting but also preserved platelets and showed higher residual platelet aggregation. This was tested in an aggressive ex vivo model of hemodialysis due to the frequent re-circulation of blood and lack of endothelial cells. This supports testing abelacimab in patients whose blood is exposed to artificial surfaces, particularly in hemodialysis.

## Electronic supplementary material

Below is the link to the electronic supplementary material.

Supplementary Material 1

## Data Availability

The article’s data will be shared on reasonable request to the corresponding author.

## References

[1] Olesen JB, Lip GY, Kamper AL, Hommel K, Kober L, Lane DA, Lindhardsen J, Gislason GH, Torp-Pedersen C (2012) Stroke and bleeding in atrial fibrillation with chronic kidney disease. N Engl J Med 367:625–635. 10.1056/NEJMoa1105594 22894575 10.1056/NEJMoa1105594

[2] Konigsbrugge O, Meisel H, Beyer A, Schmaldienst S, Klauser-Braun R, Lorenz M, Auinger M, Kletzmayr J, Hecking M, Winkelmayer WC, Lang I, Pabinger I, Saemann M, Ay C (2021) Anticoagulation use and the risk of stroke and major bleeding in patients on hemodialysis: from the VIVALDI, a population-based prospective cohort study. J Thromb Haemost 19:2984–2996. 10.1111/jth.15508 34418291 10.1111/jth.15508

[3] European Best Practice Guidelines Expert Group on Hemodialysis ERA, Section V (2002) Chronic intermittent haemodialysis and prevention of clotting in the extracorporal system. Nephrol Dial Transpl 17(Suppl 7):63–71. 10.1093/ndt/17.suppl_7.63 10.1093/ndt/17.suppl_7.63 12386229

[4] Puy C, Rigg RA, McCarty OJ (2016) The hemostatic role of factor XI. Thromb Res 141(Suppl 2):S8–S. 10.1016/S0049-3848(16)30354-1 27207433 10.1016/S0049-3848(16)30354-1 PMC6135087

[5] Lowenberg EC, Meijers JC, Monia BP, Levi M (2010) Coagulation factor XI as a novel target for antithrombotic treatment. J Thromb Haemost 8:2349–2357. 10.1111/j.1538-7836.2010.04031.x 20727068 10.1111/j.1538-7836.2010.04031.x

[6] Peyvandi F, Palla R, Menegatti M, Siboni SM, Halimeh S, Faeser B, Pergantou H, Platokouki H, Giangrande P, Peerlinck K, Celkan T, Ozdemir N, Bidlingmaier C, Ingerslev J, Giansily-Blaizot M, Schved JF, Gilmore R, Gadisseur A, Benedik-Dolnicar M, Kitanovski L, Mikovic D, Musallam KM, Rosendaal FR (2012) European Network of Rare Bleeding Disorders G. Coagulation factor activity and clinical bleeding severity in rare bleeding disorders: results from the European Network of rare bleeding disorders. J Thromb Haemost 10:615–621. 10.1111/j.1538-7836.2012.04653.x 22321862 10.1111/j.1538-7836.2012.04653.x

[7] Bolton-Maggs PH, Patterson DA, Wensley RT, Tuddenham EG (1995) Definition of the bleeding tendency in factor XI-deficient kindreds–a clinical and laboratory study. Thromb Haemost 73:194–202 7792729

[8] Duga S, Salomon O (2013) Congenital factor XI deficiency: an update. Semin Thromb Hemost 39:621–631. 10.1055/s-0033-1353420 23929304 10.1055/s-0033-1353420

[9] Nopp S, Kraemmer D, Ay C (2022) Factor XI inhibitors for Prevention and treatment of venous thromboembolism: a review on the Rationale and Update on current evidence. Front Cardiovasc Med 9:903029. 10.3389/fcvm.2022.903029 35647061 10.3389/fcvm.2022.903029 PMC9133368

[10] Ali AE, Becker RC (2024) Factor XI: structure, function and therapeutic inhibition. J Thromb Thrombolysis. 10.1007/s11239-024-02972-5 38622277 10.1007/s11239-024-02972-5 PMC11645426

[11] Walsh M, Bethune C, Smyth A, Tyrwhitt J, Jung SW, Yu RZ, Wang Y, Geary RS, Weitz J, Bhanot S, Investigators CS (2022) Phase 2 study of the factor XI antisense inhibitor IONIS-FXI(rx) in patients with ESRD. Kidney Int Rep 7:200–209. 10.1016/j.ekir.2021.11.011 35155859 10.1016/j.ekir.2021.11.011 PMC8820988

[12] Lorentz CU, Tucker EI, Verbout NG, Shatzel JJ, Olson SR, Markway BD, Wallisch M, Ralle M, Hinds MT, McCarty OJT, Gailani D, Weitz JI, Gruber A (2021) The contact activation inhibitor AB023 in heparin-free hemodialysis: results of a randomized phase 2 clinical trial. Blood 138:2173–2184. 10.1182/blood.2021011725 34086880 10.1182/blood.2021011725 PMC8641100

[13] Koch AW, Schiering N, Melkko S, Ewert S, Salter J, Zhang Y, McCormack P, Yu J, Huang X, Chiu YH, Chen Z, Schleeger S, Horny G, DiPetrillo K, Muller L, Hein A, Villard F, Scharenberg M, Ramage P, Hassiepen U, Cote S, DeGagne J, Krantz C, Eder J, Stoll B, Kulmatycki K, Feldman DL, Hoffmann P, Basson CT, Frost RJA, Khder Y (2019) MAA868, a novel FXI antibody with a unique binding mode, shows durable effects on markers of anticoagulation in humans. Blood 133:1507–1516. 10.1182/blood-2018-10-880849 30692123 10.1182/blood-2018-10-880849

[14] Samaha E, Schwameis M, Schranz S, Watschinger B, Buchmuller A, Jilma B (2019) Acetylsalicylic acid decreases clotting in combination with enoxaparin during haemodialysis in vitro. Nephrol Dial Transpl 34:509–515. 10.1093/ndt/gfy229 10.1093/ndt/gfy229 30053218

[15] Spiel AO, Bartko J, Schwameis M, Firbas C, Siller-Matula J, Schuetz M, Weigl M, Jilma B (2011) Increased platelet aggregation and in vivo platelet activation after granulocyte colony-stimulating factor administration. A randomised controlled trial. Thromb Haemost 105:655–662. 10.1160/TH10-08-0530 21301783 10.1160/TH10-08-0530

[16] Schneditz D (2011) TMP revisited: the importance of plasma colloid osmotic pressure in high-flux dialyzers. Nephrol Dial Transpl 26:411–413. 10.1093/ndt/gfq784 10.1093/ndt/gfq784 21273239

[17] Kim JC, Garzotto F, Cruz DN, Clementi A, Nalesso F, Kim JH, Kang E, Kim HC, Ronco C (2012) Computational modeling of effects of mechanical shaking on hemodynamics in hollow fibers. Int J Artif Organs 35:301–307. 10.5301/ijao.5000094 22505197 10.5301/ijao.5000094

[18] Kaw D, Malhotra D (2006) Platelet dysfunction and end-stage renal disease. Semin Dial 19:317–322. 10.1111/j.1525-139X.2006.00179.x 16893410 10.1111/j.1525-139X.2006.00179.x

[19] Ambuhl PM, Wuthrich RP, Korte W, Schmid L, Krapf R (1997) Plasma hypercoagulability in haemodialysis patients: impact of dialysis and anticoagulation. Nephrol Dial Transpl 12:2355–2364. 10.1093/ndt/12.11.2355 10.1093/ndt/12.11.2355 9394323

[20] Baaten C, Sternkopf M, Henning T, Marx N, Jankowski J, Noels H (2021) Platelet function in CKD: a systematic review and Meta-analysis. J Am Soc Nephrol. 10.1681/ASN.2020101440 33941607 10.1681/ASN.2020101440 PMC8425648

[21] Sood MM, Komenda P, Sood AR, Rigatto C, Bueti J (2009) The intersection of risk and benefit: is warfarin anticoagulation suitable for atrial fibrillation in patients on hemodialysis? Chest 136:1128–1133. 10.1378/chest.09-0730 19809054 10.1378/chest.09-0730

[22] Decousus H, Tapson VF, Bergmann JF, Chong BH, Froehlich JB, Kakkar AK, Merli GJ, Monreal M, Nakamura M, Pavanello R, Pini M, Piovella F, Spencer FA, Spyropoulos AC, Turpie AG, Zotz RB, Fitzgerald G, Anderson FA, Investigators I (2011) Factors at admission associated with bleeding risk in medical patients: findings from the IMPROVE investigators. Chest 139:69–79. 10.1378/chest.09-3081 20453069 10.1378/chest.09-3081

[23] Potpara TS, Ferro CJ, Lip GYH (2018) Use of oral anticoagulants in patients with atrial fibrillation and renal dysfunction. Nat Rev Nephrol 14:337–351. 10.1038/nrneph.2018.19 29578207 10.1038/nrneph.2018.19

[24] Hellenbart EL, Faulkenberg KD, Finks SW (2017) Evaluation of bleeding in patients receiving direct oral anticoagulants. Vasc Health Risk Manag 13:325–342. 10.2147/VHRM.S121661 28860793 10.2147/VHRM.S121661 PMC5574591

[25] Mavrakanas TA, Garlo K, Charytan DM (2020) Apixaban versus no anticoagulation in patients undergoing long-term Dialysis with Incident Atrial Fibrillation. Clin J Am Soc Nephrol 15:1146–1154. 10.2215/CJN.11650919 32444398 10.2215/CJN.11650919 PMC7409754

[26] Feldberg J, Patel P, Farrell A, Sivarajahkumar S, Cameron K, Ma J, Battistella M (2019) A systematic review of direct oral anticoagulant use in chronic kidney disease and dialysis patients with atrial fibrillation. Nephrol Dial Transpl 34:265–277. 10.1093/ndt/gfy031 10.1093/ndt/gfy031 29509922

[27] Li W, Zhou Y, Chen S, Zeng D, Zhang H (2022) Use of non-vitamin K antagonists oral anticoagulants in atrial fibrillation patients on dialysis. Front Cardiovasc Med 9:1005742. 10.3389/fcvm.2022.1005742 36176998 10.3389/fcvm.2022.1005742 PMC9513185

[28] Reinecke H, Brand E, Mesters R, Schabitz WR, Fisher M, Pavenstadt H, Breithardt G (2009) Dilemmas in the management of atrial fibrillation in chronic kidney disease. J Am Soc Nephrol 20:705–711. 10.1681/ASN.2007111207 19092127 10.1681/ASN.2007111207

[29] Buller HR, Bethune C, Bhanot S, Gailani D, Monia BP, Raskob GE, Segers A, Verhamme P, Weitz JI, Investigators F-AT (2015) Factor XI antisense oligonucleotide for prevention of venous thrombosis. N Engl J Med 372:232–240. 10.1056/NEJMoa1405760 25482425 10.1056/NEJMoa1405760 PMC4367537

[30] Weitz JI, Bauersachs R, Becker B, Berkowitz SD, Freitas MCS, Lassen MR, Metzig C, Raskob GE (2020) Effect of Osocimab in preventing venous thromboembolism among patients undergoing knee arthroplasty: the FOXTROT Randomized Clinical Trial. JAMA 323:130–139. 10.1001/jama.2019.20687 31935028 10.1001/jama.2019.20687 PMC6990695

[31] Verhamme P, Yi BA, Segers A, Salter J, Bloomfield D, Buller HR, Raskob GE, Weitz JI, Investigators A-T (2021) Abelacimab for Prevention of venous thromboembolism. N Engl J Med 385:609–617. 10.1056/NEJMoa2105872 34297496 10.1056/NEJMoa2105872

[32] Pfeffer MA, Kohs TCL, Vu HH, Jordan KR, Wang JSH, Lorentz CU, Tucker EI, Puy C, Olson SR, DeLoughery TG, Hinds MT, Keshari RS, Gailani D, Lupu F, McCarty OJT, Shatzel JJ (2024) Factor XI inhibition for the Prevention of Catheter-Associated thrombosis in patients with Cancer Undergoing Central Line Placement: a phase 2 clinical trial. Arterioscler Thromb Vasc Biol 44:290–299. 10.1161/ATVBAHA.123.319692 37970718 10.1161/ATVBAHA.123.319692 PMC10877270

[33] Ashman N, Macey MG, Fan SL, Azam U, Yaqoob MM (2003) Increased platelet-monocyte aggregates and cardiovascular disease in end-stage renal failure patients. Nephrol Dial Transpl 18:2088–2096. 10.1093/ndt/gfg348 10.1093/ndt/gfg348 13679485

[34] Migliori M, Cantaluppi V, Scatena A, Panichi V (2017) Antiplatelet agents in hemodialysis. J Nephrol 30:373–383. 10.1007/s40620-016-0367-5 27928736 10.1007/s40620-016-0367-5

[35] Weitz JI, Tanko LB, Floege J, Fox KAA, Bhatt DL, Thadhani R, Hung J, Pap AF, Kubitza D, Winkelmayer WC, Investigators C (2024) Anticoagulation with osocimab in patients with kidney failure undergoing hemodialysis: a randomized phase 2 trial. Nat Med 30:435–442. 10.1038/s41591-023-02794-7 38365952 10.1038/s41591-023-02794-7 PMC10878964

[36] Winkelmayer WC, Lensing AWA, Thadhani RI, Mahaffey KW, Walsh M, Pap AF, Willmann S, Thelen K, Hodge S, Solms A, Ingham SJM, Eikelboom J (2024) Investigators R-T. A phase II randomized controlled trial evaluated antithrombotic treatment with fesomersen in patients with kidney failure on hemodialysis. Kidney Int 106:145–153. 10.1016/j.kint.2024.02.024 38537676 10.1016/j.kint.2024.02.024

[37] Meyer AD, Thorpe CR, Fraker T, Cancio T, Rocha J, Willis RP, Cap AP, Gailani D, Shatzel JJ, Tucker EI, McCarty OJT (2023) Factor XI inhibition with heparin reduces clot formation in simulated Pediatric extracorporeal membrane oxygenation. ASAIO J 69:1074–1082. 10.1097/MAT.0000000000002048 37801726 10.1097/MAT.0000000000002048 PMC10841048

[38] Ashby D, Borman N, Burton J, Corbett R, Davenport A, Farrington K, Flowers K, Fotheringham J, Andrea Fox RN, Franklin G, Gardiner C, Martin Gerrish RN, Greenwood S, Hothi D, Khares A, Koufaki P, Levy J, Lindley E, Macdonald J, Mafrici B, Mooney A, Tattersall J, Tyerman K, Villar E, Wilkie M (2019) Renal Association Clinical Practice Guideline on Haemodialysis. BMC Nephrol 20:379. 10.1186/s12882-019-1527-3 31623578 10.1186/s12882-019-1527-3 PMC6798406

[39] Natale P, Palmer SC, Ruospo M, Longmuir H, Dodds B, Prasad R, Batt TJ, Jose MD, Strippoli GF (2024) Anticoagulation for people receiving long-term haemodialysis. Cochrane Database Syst Rev 1:CD011858. 10.1002/14651858.CD011858.pub2 38189593 10.1002/14651858.CD011858.pub2 PMC10772979

[40] Novacek G, Kapiotis S, Jilma B, Quehenberger P, Michitsch A, Traindl O, Speiser W (1997) Enhanced blood coagulation and enhanced fibrinolysis during hemodialysis with prostacyclin. Thromb Res 88:283–290. 10.1016/s0049-3848(97)00255-7 9526948 10.1016/s0049-3848(97)00255-7

[41] Sagedal S, Hartmann A, Sundstrom K, Bjornsen S, Fauchald P, Brosstad F (1999) A single dose of dalteparin effectively prevents clotting during haemodialysis. Nephrol Dial Transpl 14:1943–1947. 10.1093/ndt/14.8.1943 10.1093/ndt/14.8.1943 10462275

[42] Xu L, Sun Y, Wang S, Li C, Mao Y (2023) Anti-xa level monitoring of low-molecular-weight heparin during intermittent venovenous hemofiltration. Ann Hematol 102:2251–2256. 10.1007/s00277-023-05290-7 37395763 10.1007/s00277-023-05290-7 PMC10344977

