# Supplementary material for: The factor XI/XIa antibody abelacimab combined with enoxaparin inhibits filter clotting in hemodialysis circuits ex vivo
Source: J Thromb Thrombolysis. 2024 Nov 16;57(8):1339–48. doi: 10.1007/s11239-024-03059-x (PMC11645315; doi:10.1007/s11239-024-03059-x)

**Table S1.** List of inclusion/exclusion criteria

Inclusion criteria:

- Healthy male and female volunteers aged >18 and < 60 years
- Signed informed consent was obtained before any study-related activities (study activities are any procedures that would not have been performed during normal management of the subject)
- Ability to comprehend the full nature and purpose of the study, including possible risks and side effects; ability to cooperate with the investigator and to comply with the requirements of the entire study
- Normal findings in medical history and physical examination unless the investigator considers an abnormality to be clinically irrelevant

Exclusion criteria:

- Known Hepatitis C, Hepatitis B, or Human immunodeficiency virus
- Blood donations during 1 month before this study
- Anemia (defined as <12g/dl Hb for females and <13,5g/dl Hb for males)
- Relevant history of renal, hepatic, gastrointestinal, cardiovascular, respiratory, skin, hematological, endocrine, inflammatory, or neurological diseases that may interfere with the aim of the study.
- Use of medication 2 weeks before the start of the study, which the investigator considers may affect the validity of the study except for hormonal contraception in female subjects
- Pregnancy (positive pregnancy test on the day of blood donation) or lactation

**Table S2.** Laboratory parameters of the circuits.

|  | Group | | | | | |  |
| --- | --- | --- | --- | --- | --- | --- | --- |
|  | Enoxaparin | | | Enoxaparin + Abelacimab | | |  |
|  | Median | P 25 | P 75 | Median | P 25 | P 75 |  |
| Platelets at 0min | 140 | 126 | 166 | 152 | 135 | 170 | 0.481 |
| Platelets at EoC | 106 | 95 | 119 | 135 | 127 | 149 | 0.035 |
| Potassium at 0 min | 3.25 | 3.20 | 3.30 | 3.25 | 3.20 | 3.40 | 0.796 |
| Potassium at EoC | 2.90 | 2.90 | 3.00 | 2.90 | 2.90 | 2.90 | 0.436 |

P25 … 25^th^ percentile; P75 … 75^th^ percentile; EoC ... End of Circuit

**Figure SF1.** Experimental setup with the Nikkiso Hemodialysis devices (DBB 07).


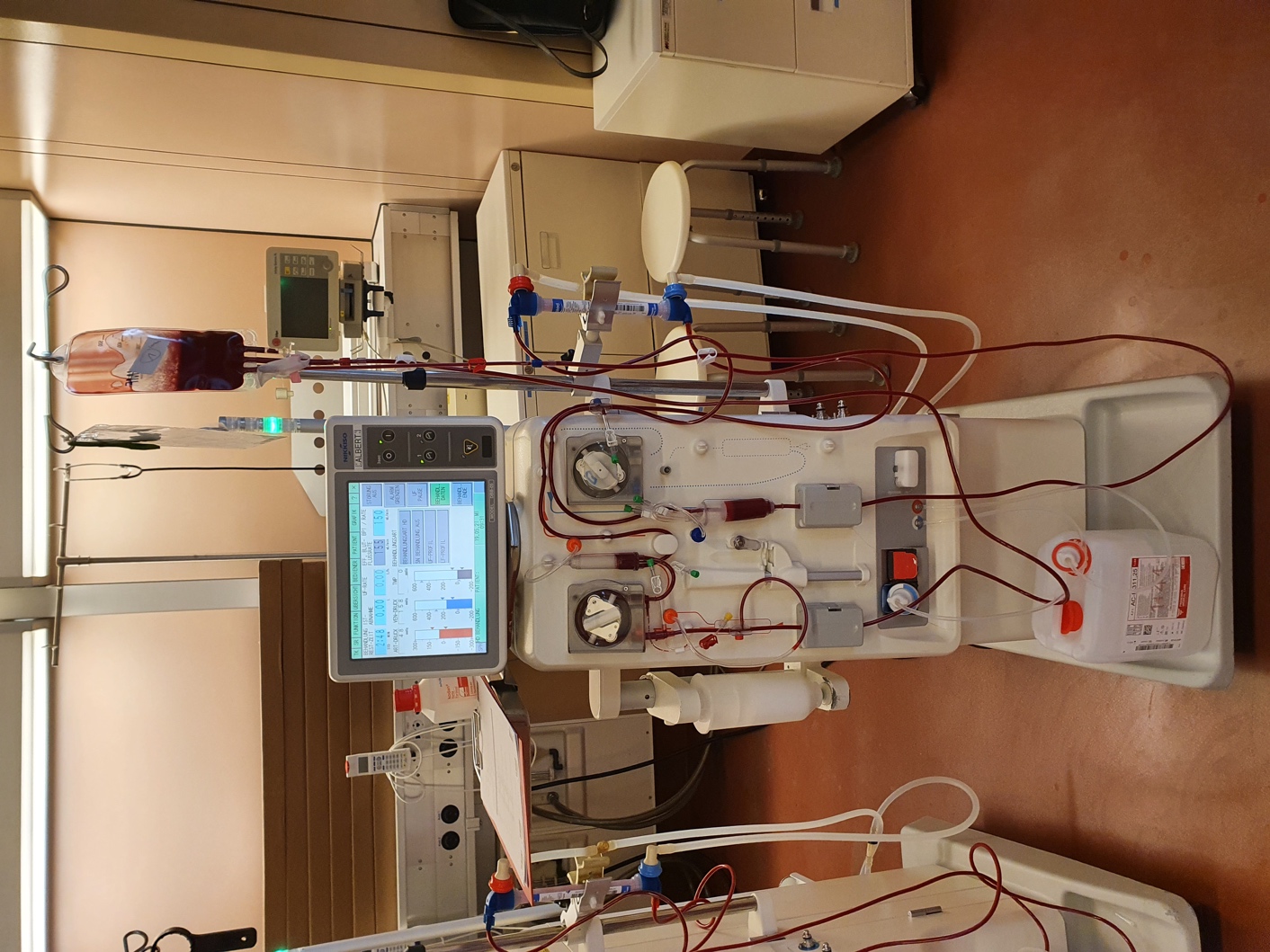

Supplement: Supplementary file 1 — Supplementary Material 1 [file 11239_2024_3059_MOESM1_ESM.docx]
